# Efficacy of alginate-coated gold nanoparticles against antibiotics-resistant *Staphylococcus* and *Streptococcus* pathogens of acne origins

**DOI:** 10.1515/biol-2022-1045

**Published:** 2025-02-25

**Authors:** Hanan A. Abbas, Ali A. Taha, Ghassan M. Sulaiman, Amer Al Ali, Humood Al Shmrany, Haralambos Stamatis, Hamdoon A. Mohammed, Riaz A. Khan

**Affiliations:** Division of Biotechnology, Department of Applied Sciences, University of Technology, Baghdad, Iraq; Department of Medical Laboratory Sciences, College of Applied Medical Sciences, University of Bisha, 255, Bisha, 67714, Saudi Arabia; Department of Medical Laboratory, College of Applied Medical Sciences, Prince Sattam Bin Abdulaziz University, Alkharj, 11942, Saudi Arabia; Department of Biological Applications and Technology, University of Ioannina, Ioannina, Greece; Department of Medicinal Chemistry and Pharmacognosy, College of Pharmacy, Qassim University, Qassim, 51452, Saudi Arabia

**Keywords:** nanoparticles, deactivation, anti-adhesion, anti-biofilm, *Staphylococcus*, *Streptococcus*

## Abstract

Acne is a serious multifactorial inflammatory disease that leads to significant and long-lasting changes. The widespread occurrence of bacterial acne and the excessive use of antibiotics to treat it have increased resistance to antibiotic treatment and led researchers to seek and develop newer antimicrobial agents suitable for various medical purposes. In this study, alginate-coated gold nanoparticles (GANPs), synthesized by the previously reported known method, using sodium alginate and gold salt, investigated the efficacy of the GANPs against various clinical isolates of *Staphylococcus*, i.e., *Staphylococcus aureus*, *Staphylococcus lentus*, *Staphylococcus haemolyticus*, and *Streptococcus* *thoraltensis*, which were all obtained from patients suffering from acne conditions. The results showed that the GANPs had antibacterial efficacy against all the acne-isolated bacteria. The GANP activity against bacterial resistance suggested that metal-based nanoparticulate materials are a promising alternative for treating multidrug-resistant microorganisms.

## Graphical abstract

**Figure:**
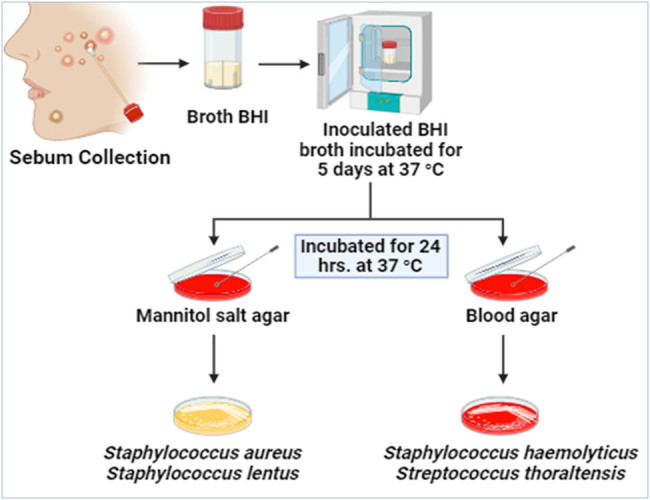

## 1 Introduction

Acne, a chronic, multifactorial, immune-mediated inflammatory disease of the pilosebaceous unit has a high global incidence. Acne primarily affects the face, shoulder, upper chest skin regions, and back [1]. The resulting appearance from the acne condition may lead to anxiety, diminished self-esteem, and depression [2]. Increased sebum production leads to follicular keratinization of the pilosebaceous glands causing acne. Moreover, inflammation promotes and increases the growth of bacteria, which are crucial in the development of acne, since it has several virulent factors and acquired antibiotic resistance. Acne pathogenesis is caused by a variety of factors. In addition to increased sebum production, abnormalities in the microbiota which lead to bacterial colonization, the body part’s hormonal changes, and alterations in immunological processes linked to inflammation cause acne [3]. The aerobic Gram-positive bacteria from genus *Staphylococcus* and *Streptococcus* are indulged in pathogenesis. The bacterial species *Staphylococcus aureus*, *Staphylococcus lentus*, *Staphylococcus haemolyticus*, and *Streptococcus thoraltensis* primarily take part in skin infection [4,5,6]. Numerous studies have linked the presence of these bacterial species to specific diseased conditions of the skin, and the instance is more common in compromised individuals than in healthy subjects. The bacterial species form biofilms as part of their infectious methodology [7,8]. The biofilm also exists as a polymicrobial structure composed of distinct populations of *Staphylococcus* and *Streptococcus* species. The phenotypic biofilms have a definite role in enhanced antibiotic resistance leading to the nullification of therapy and improvements in skin conditions. Interestingly, within the pilosebaceous unit, a biofilm matrix functions as a biological adhesive, thereby physically restricting the flow of sebum into the infundibulum to promote the formation of comedones, with retention and accumulation of corneocytes in the lumen. As a result, the plug formed in the keratinaceous layer triggers inflammation [9,10].

Acne control and treatment have involved the use of systemic and topical antibiotics, which include treatments with chemical peels, light amplification by the stimulated emission of radiation, and hormone therapy. Nevertheless, these treatments are accompanied by several side effects, such as increased microbial skin irritation, stomatitis, and resistance to antibiotics, in particular at a stage when bacterial cells start to adhere and form biofilms. Therefore, there is still a critical requirement for alternative therapies for combating acne [11].

Medical nanobiotechnology has taken precedence in putting forward suggestions toward a novel treatment regime for several physiological conditions, including skin conditions, owing to their easy and focused outreach, dose control, site specificity, and enhanced bioaction. The size-dependent properties of the nano-scale material have made them a unique and favorable tool fit for biological and pharmacological bioactions [12]. The naked and surface-coated metallic nanoparticles (MNPs) sourced from gold, silver, and other common metals, along with their corresponding oxides, have been recommended as potential antibacterial agents. The MNPs possess properties that enable them to inhibit the activity of the miniscule bacteria broth responsible for infection. Additionally, the MNPs have been reported to utilize several different mechanisms to contain the bacterial cells, thereby leading to either bacterial cell dysfunction or infectious cell death [13].

After the trend of applying silver nanoparticles (AgNPs) as an antibacterial agent, the concurrent use of MNPs, especially gold nanoparticles (AuNPs), has garnered significant attention in the field of nanobiotechnology. The versatility of AuNPs is owing to their ability to adjust through various maneuverable *modus operandi*, which is possible because of their nano-scale size, shape, surface area, and scope for the choicest surface chemistry modifications through a set of chemical attachment/transformation procedures. This versatility has produced the desired outcome at different levels of bioactivity through the prepared nanoparticulate formulations. Several research groups have regarded AuNPs as among the most versatile and nearly universal antimicrobial agents because of their non-toxic nature and harmless properties. A number of different circumstantial factors of biotic and abiotic nature, as well as the properties of the AuNPs, have supported the selection of AuNPs as the choice of nanomaterial for antimicrobial drug development at the nano-scale level [14,15].

Recent studies have highlighted the use of AuNPs in the photothermal treatment of acne, which is effective without needing the use of antibiotics [16]. Studies have shown that photothermal therapy using AuNPs effectively treated various types of acne, including comedones, bacterial acne, and inflammatory lesions in a short time [17]. Additionally, AuNPs have received attention due to their biocompatibility, which allows them to interact well with living organisms at the cellular level [18,19,20]. The preparative methods of the nano-scale drug formulation, the favorable variability in starting salt concentration, the reaction conditions, preparative media, use of chemical modifiers of surfactant and emulsifiers for controlling the produced nano-scale entities, the open choice of produced MNPs’ coating material that is adjustable and compatible with the MNPs, and the surface reactions leading to the desired size, shape, size distribution, anti-flocculation, and inhibition to the coated nanoparticles aggregation have made AuNPs the desired nano-scale entities for the further biochemical and biological activity evaluations [21,22,23,24].

The use of natural products, on a whole, has scored precedence over hazardous and non-eco-friendly chemical materials. The abundant natural entities, owing to their ease of handling, affordability, and biocompatibility, have been the first choice of raw materials for nano-entities preparation [25,26]. Sodium alginate, a marine-sourced polysaccharide, is non-toxic, biocompatible, and biodegradable owing to its prevalence of hydroxyl groups in its structure. Several free hydroxyl and carboxyl groups form their backbone, which play major roles in the bioreduction mechanism of the metal salt, including the gold precursor [27]. Mechanistically, the hydroxyl groups present in the alginate provide electrons to nullify the positively charged Au^3+^ ions. This process converts them into Au^0^ and generates basic nascent AuNPs, which further self-group to form the AuNPs. Furthermore, the interactions between the developing AuNPs and the alginate’s functional groups make it convenient to limit the size of the forming AuNPs with its coating which prohibits flocculation [28]. Previous studies have demonstrated that the shape and size of the AuNPs are important deciding factors for their biological activity, such as transport, required accumulation at the site, and cell and bacteria death. For the body, the kidneys have shown rapid excretion of the NPs under 6 nm in size from the body while the biological half-life of these NPs in the 10–100 nm size range has been found to be increased with an increase in size. Notwithstanding this, the AuNPs smaller than 200 nm have also been utilized for bioactivity evaluation purposes [14,29,30].

The current study aimed to fill in the gaps related to the control of the resistant microbial pathogens of acne using alginate-coated gold nanoparticles (GANPs). The nanoparticles were produced through the reduction of aurum (Au) salt using sodium alginate as both the bio-reducing agent and the covering material in our earlier work [31]. This study tested the antibacterial efficacy of the produced GANPs against various *Staphylococcus* species, preferably the face. *Staph aureus*, *Staph lentus*, *Staph haemolyticus*, and *S. thoraltensis* were isolated from human acne for antibacterial bio-testing and evaluation of any inherent toxicity to confirm the safety of the GANPs as a treatment tool. It explored the potential of the alginate-coated AuNPs as a therapeutic agent for acne conditions.

## 2 Materials and methods

### 2.1 Chemicals and reagents

Chloroauric acid (HAuCl_4_·3H_2_O), sodium alginate, and crystal violet stain were purchased from Sigma Aldrich Chemical Co. (St. Louis, MO, USA). Brain heart infusion (BHI) broth, Muller–Hinton agar (MHA). Muller–Hinton broth (MHB) medium, mannitol salt agar (MSA), blood agar medium, and trypticase soy broth (TSB) were purchased from HiMedia (India). All other reagents and chemicals were of analytical grade.

### 2.2 Isolation of bacterial acne

Ninety acne patients, suffering from mild, moderate, and severe acne in their different face regions, i.e., jaw, forehead, temple, chin, and cheeks, with ages 15–25 years, were enrolled in this study from different private clinics and volunteers from various governorates. The medical city in Baghdad was approached for authorization, and the study was approved in accordance with Reference No. 4712 ASBT 8/11/2022. Written consent was given by each participant. Also, we conducted all experiments in accordance with the guidelines set forth by the Food and Drug Administration and the National Institute of Health.

**Figure 1:**
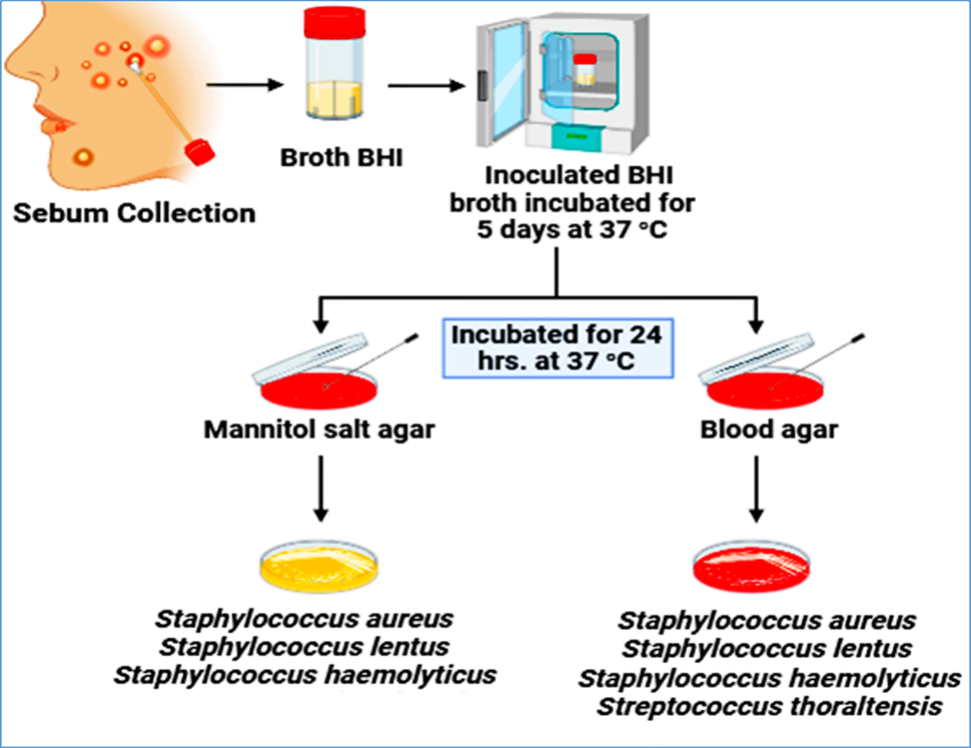
Collection of bacterial acne isolates in BHI broth cultured on blood agar and MSA under aerobic conditions.


The divisions of acne lesions were categorized into three groups depending on their distribution over half of the face: subtle acne number (0–5), intermediate acne number (6–20), and acute acne number (21–50) [32]. The bacteria that were discovered on the acne lesion were eliminated using an alcohol pad. The sebum was then gently extracted from the acne lesion using a small, sharp lancet and a hand swab. The tube holding 5 mL of BHI broth was employed as a transport medium for the swab. The tubes were incubated at 37°C for 5 days [33]. The medium that tested positive for BHI was found to be turbid, indicating the presence of bacterial growth, and the material was utilized for the study (Figure 1). All the samples were collected by our laboratory in accordance with the hospital protocols, and the standard operating procedures for sample collection, packing, and transport of the samples. The standard good clinical practices (GLP) were followed in the handling of the samples at all stages of the work [34].

**Informed consent:** Informed consent has been obtained from all individuals included in this study.
**Ethical approval:** The research related to human use has been complied with all the relevant national regulations and institutional policies and in accordance with the tenets of the Helsinki Declaration and has been approved by the authors’ institutional review board or equivalent committee.

### 2.3 Identification of bacterial isolates

Blood agar medium and MSA were processed following the manufacturer’s instructions and further utilized as culturing and purifying media. Blood agar was utilized as an enrichment medium to grow different bacterial species and differentiate the bacteria based on their hemolytic features [35]. The MSA was used to isolate and diagnose the *Staphylococcus* species [36]. The samples were grown aerobically and incubated for 24 h at 37°C. The process of purifying the colony was repeated twice. Following the homogenization of the media, the isolates were identified using bacterial cell types (spores and Gram staining) and colony morphology (shape, color, surface, edge, and elevation colonies). The identification process was subsequently confirmed using the VITEK 2 system (VITEK, Biomérieux, Marcy-l’Etoile, France). For analysis of their antibacterial activity, four acne-isolated bacteria with various colony morphologies were chosen (Figure 1).

### 2.4 Preparation of GANPs

**Figure 2:**
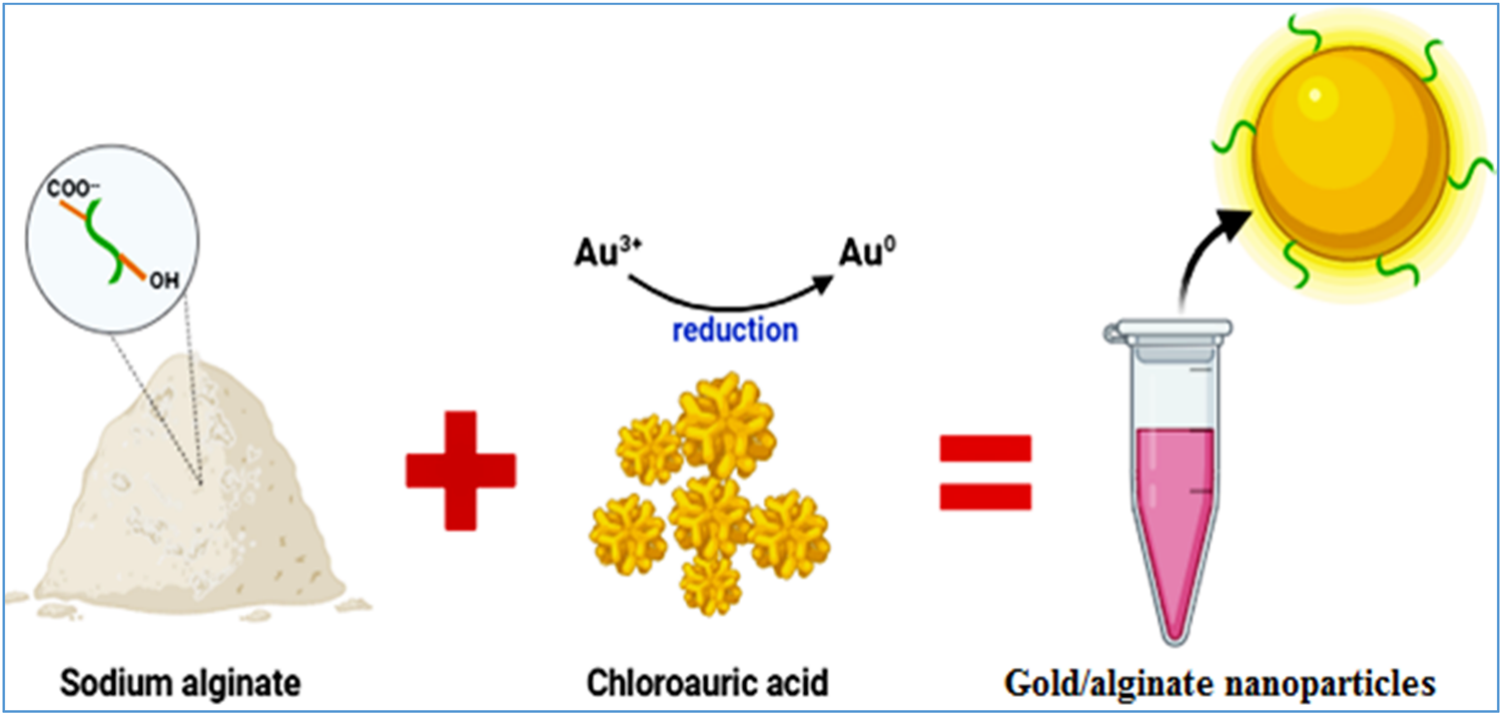
Chemical reduction of chloroauric acid by sodium alginate to synthesized alginate-coated AuNPs.

To prepare GANPs, 6.79 mg of HAuCl_4_ was dissolved in 10 mL of distilled water (DW), yielding an aqueous solution of 2 mM HAuCl_4_. Five milliliters of sodium alginate aqueous solution (1 g/1 L, DW, M.W. 12–40 KD) were added to this mixture. To finish the reduction of gold ions and create GANPs [37], the reaction mixture was heated at 80°C for 40 min, allowed to cool, and collected (Figure 2).

### 2.5 Characterization of GANPs

According to our recently published work [31], Ultraviolet–visible (UV-Vis) spectrophotometry was used to characterize the GANPs and their starting raw materials (HAuCl_4_ and sodium alginate), wherein each sample was dissolved in DW. Then, 1.5 mL of each was placed in a cuvette, which was measured with an absorbance range of 200–800 nm [38].

Scanning electron microscopy (SEM) was used to determine the size, morphology, surface morphology, and distribution of the samples. Thin films were prepared by just dropping the sample on a glass slide and then allowing them to dry; after that, they were metalized for non-conductive samples (sodium alginate and HAuCl_4_) and then examined [39] by an Inspect F50 SEM from FEI Company (Netherlands).

Fourier transform infrared spectrophotometry (FT-IR) was used for the interactions, the role of functional groups in each sample on nanoparticle formation, and the investigation of capping agents. The solution of each sample was mixed with potassium bromide at a ratio of 1:100 and then analyzed by FT-IR Perkin Elmer (USA) within a wavelength range of 500–4,000 nm.

X-ray diffraction (Aeris, Netherlands) was used to determine if the material is amorphous or crystalline and the size and peak intensity of each sample. It is commonly used to investigate the structure of MNPs by penetrating the material with X-rays. On a glass slide, a thin layer of each sample was placed, and the sample was analyzed at an operating voltage of 40 kV and scan range between 10° and 90° at 2*θ* angles [40].

Energy-dispersive X-ray (EDX) spectroscopy investigated the chemical composition and elemental percentage. Thin films were prepared by just dropping the samples on glass slides and then metalizing for non-conductive samples (sodium alginate and HAuCl_4_). After that, they were examined [41] by EDX (Axia, Netherlands).

### 2.6 Determination of the antibacterial activity of GANPs

The antibacterial activity methods were done according to the guidelines established by the Clinical and Laboratory Standard Institute [42].

#### 2.6.1 Agar well-diffusion method

The antibacterial effectiveness of GANPs against the acne-isolated bacteria *Staph aureus, Staph haemolyticus*, *Staph lentus*, and *S. thoraltensis* was evaluated using the agar well-diffusion method. The MHA plate was inoculated by equally covering the whole surface of the agar with a bacterial suspension previously prepared by picking (4–5) colonies of each bacterial isolate from the original culture, and they were suspended in the test tube containing 4 mL of normal saline solution. The standard turbidity solution McFarland standard was made from sulfuric acid (H_2_SO_4_) and barium chloride (BaCl_2_·2H_2_O) equal to 1.5 × 10^8^ CFU/mL, according to protocol’s instructions [43]. Next, a 6-mm-diameter well was aseptically punched using a conventional cork borer. The wells received a single dose of 80 µL from GANPs at four different concentrations (6,400, 3,200, 1,600, and 800 µg/mL). DW served as the control. Agar plates were then incubated for 24 h at 37°C. The resultant inhibitory zones [44] were measured using digital Vernier Calipers (USA). For each treatment, three experiments were conducted.

#### 2.6.2 Minimum inhibitory concentration (MIC) assay

For the GANPs, a 96-well sterile polystyrene microplate was used to measure the MIC. First, 100 µL of MHB with two-fold dilutions of 6,400 μg/mL of GANPs was added to every well. Rows 1–10 had a series of GANP dilutions ranging from 6,400 to 12.5 µg/mL. Lastly, 100 µL of a standard of previously made bacterial suspension with 10^8^ CFU/mL was added to each well and mixed gently. There was just 200 µL of nutritional broth in the eleventh row, which was utilized as a negative control. On the other hand, the twelfth row, which served as a positive control, was shaken while being incubated at 37°C for 24 h, containing 100 µL of bacterial suspension and 100 µL of MHB. To check for antibacterial activity, 20 µL of resazurin (Sigma-Aldrich, MO, USA) was added to each well. The plates were then left to sit for an hour, and the results were read by looking at how the color of the resazurin changed [45]. Every experiment was repeated to validate the results.

#### 2.6.3 Detection of biofilm formation

Using the 96-well polystyrene microplate method and following the quantitative assessment protocol [46], biofilm developments were identified. Three to five carefully separated colonies were used to create the bacterial suspension, which was then tested by the 0.5 McFarland turbidity standard after being inoculated in 10 mL of TSB containing 0.25% glucose. Following an overnight incubation period, a new TSB medium was used to dilute the infected broth to a ratio of 1:100, and subsequent to that, 200 μL of bacterial suspension was poured into the polystyrene microplate with 96 flat bottom wells. Following a 24-h incubation period at 37°C, the broth was disposed of and washed twice with 200 μL of phosphate-buffered saline (PBS) (pH 7.3) to exclude the non-adherent cells. After fixing the bacterial biofilms with 98% methanol, the films were stained for 30 min with 0.1% (w/v) crystal violet and rinsed with DW to get rid of any leftover stains. Next, 200 µL of 33% glacial acetic acid was added. The optical density was determined using a microplate reader (BioTek, Winooski, USA) at a wavelength of 590 nm. Based on the optical density of the bacterial films, all species were categorized into three groups: non/weak, moderate, and robust, as shown in Table 1 [47]. The optical density data measured in triplicate were averaged for the results.

**Table 1:** Classification of biofilm formation

Mean OD values	Biofilm formation
<0.25	Non/weak
0.25–0.75	Moderate
≥0.75	Robust

#### 2.6.4 Anti-biofilm assay

A 96-well polystyrene microplate was used to evaluate the anti-biofilm properties of GANPs against the acne-isolated bacteria *Staph aureus*, *Staph haemolyticus*, *Staph lentus*, and *S. thoraltensis*. Each well held 180 μL of BHI broth and was seeded with 10 μL of a 10^8^ CFU/mL bacterial culture. Subsequently, 10 μL of GANPs was added at doses of 100, 150, 200, 250, 300, 350, 400, 500, 600, and 700 µg/mL. The contents of the plates were taken out and rinsed four times with PBS (pH 7.3) to get rid of any remaining free bacteria after being incubated for 24 h at 37°C. Aqueous 95% ethanol was used to fix bacterial biofilms, and 0.1% (w/v) crystal violet was used to dye. DW was used to wash it 5 times to remove any remaining stain, which was then allowed to dry. Next, 200 µL of 33% glacial acetic acid was added, and a microplate reader was used to obtain results at 590 nm after 15 min of the treatment. The absorbance was taken into account for the biofilm formations and the bacterial adherence on the surface of the nanoparticles. The average of each concentration’s triplicate reads was determined [48].

#### 2.6.5 Anti-adhesion assay

A polystyrene microplate with 96 wells was used to test GANPs’ anti-adhesion properties against the acne-isolated bacteria *Staph aureus*, *Staph haemolyticus*, *Staph lentus*, and *S. thoraltensis*. To 200 µL of bacterial culture containing 10^8^ CFU/mL, a thin layer of GANPs at concentrations of 100, 200, 600, and 700 µg/mL was added to each well. After 4 h of incubation at 37°C, DW was used to rinse it three times. Afterward, each slide was covered with 200 µL of methanol for 15 min to fix the adhering bacteria, and following the DW’s removal of the non-adherent bacteria, 200 µL of 1%, w/v crystal violet was applied to the slides and incubated for 15 min. The dyed slides were washed with DW, and after the bound dye was removed using 200 µL of (33%, w/v) glacial acetic acid, the optical density of the solubilized dye was measured at 630 nm using an automatic plate reader [49].

### 2.7 Statistical analysis

The SPSS statistical program, version IBM SPSS 29, was used, and the data were analyzed using a one-way analysis of variance (SPSS Inc., Chicago, IL, USA). The mean and standard deviation (SD) of the data were displayed. Every experiment was run in triplicate.

## 3 Results and discussion

### 3.1 Characterization of GANPs

The characterization of GANPs and their starting raw materials (HAuCl_4_ and sodium alginate) were previously reported in our published work [31]. The distribution, morphology, and size distribution were revealed by SEM, confirming the GANPs’ spherical shape and the particle size range of 19.43–37.37 nm. The Ultraviolet–visible (UV-Vis) spectrum spectrum showed the peak absorption maxima at 546 nm, which was owing to the GANPs’ localized surface plasmon resonance. The crystallite size (*D*) of GANPs was also determined using Scherer’s equation, which estimated the average size to be approximately 37.53 nm and was consistent with the SEM data. The X-ray analysis confirmed the physical state of the crystalline structure; thus, it was possible to identify the crystal formation of GANPs using this technique. Determining the size of GANPs provided information on their effectiveness and behavior as an antibacterial agent. Fourier transform infrared spectroscopy (FT-IR) was utilized to identify the interactions between the functional groups of sodium alginate and the HAuCl_4_ starting materials, which was taken as the confirmation of the completion of the reduction reaction. The E-mapping analysis of the GANPs confirmed the presence of Au, Na, O, and C. The characteristic peak of Au had a weight percentage of 19.55 while the percentages of Na, O, and C were 1.4, 45.4, and 33.8, respectively. The C is sourced from the alginate moieties that are present in GANPs.

### 3.2 Isolation and identification of acne-isolated bacteria

**Figure 3:**
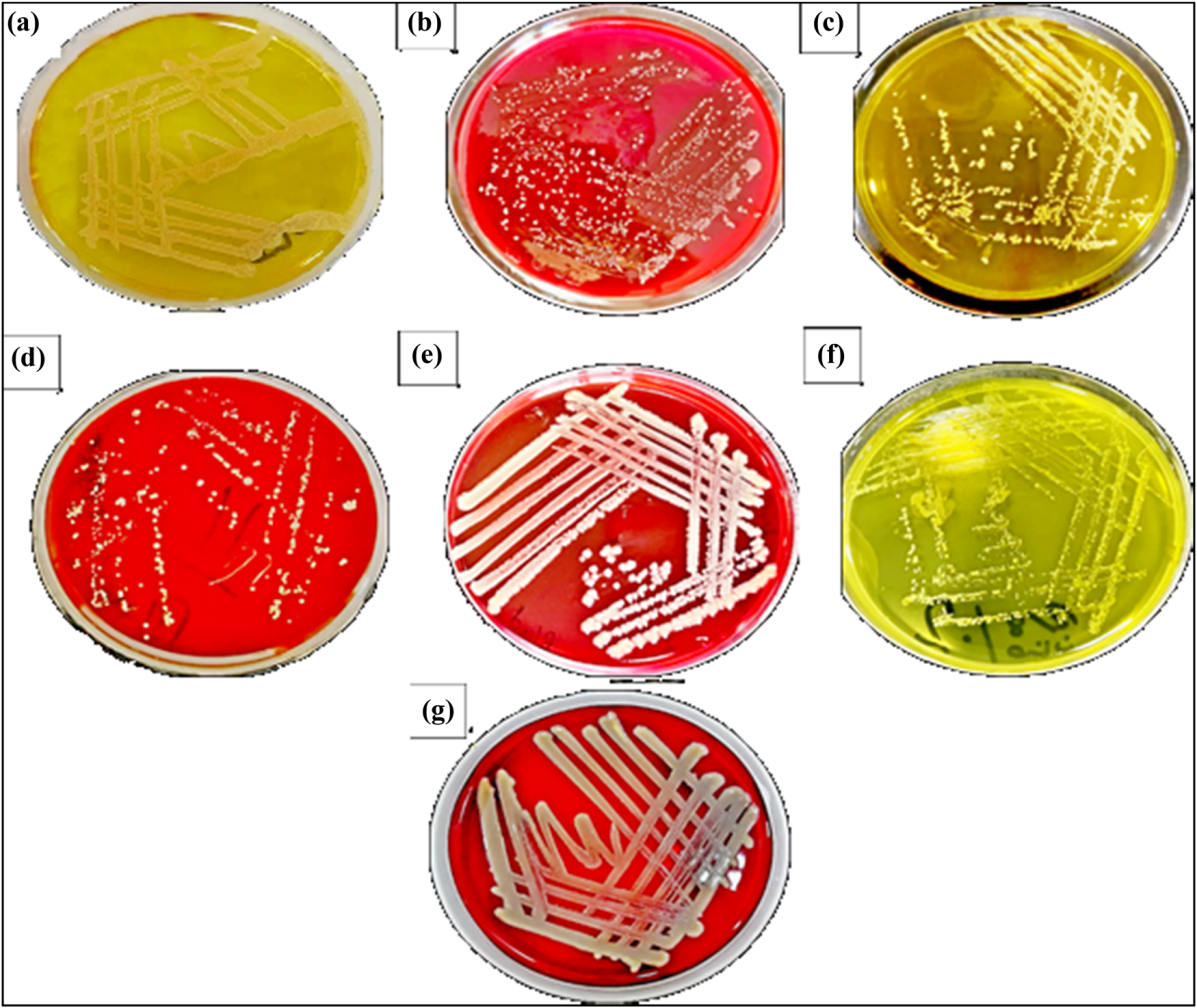
Acne-isolated bacteria on MSA and blood agar. (a and b) *Staph aureus*, (c and d) *Staph lentus*, (e and f) *Staph haemolyticus*, and (g) *S. thoraltensis* only on blood agar.

To identify the isolates of bacterial acne in addition to diagnosis with the VITEK 2 system, the isolates were cultured on blood agar to differentiate bacteria depending on their hemolytic activity and morphology and on MSA to differentiate the *Staphylococcus* species depending on their ability to grow in a high salt environment by fermenting mannitol to generate acids that change the phenol red indicator from red to yellow, as shown in Figure 3. On blood agar, *Staph aureus* appeared creamy-white and large, and formed β-hemolytic; *Staph lentus* showed white to yellow, small, and non-hemolytic characteristics; *Staph haemolyticus* showed beige to white, small, and non-hemolytic characteristics; and *S. thoraltensis* appeared as small, glossy, gray, and smooth colonies and was non-hemolytic. On MSA, *Staph aureus* appeared shiny yellow, convex, and round; *Staph lentus* appeared circular, smooth, small, and white; *Staph haemolyticus* appeared convex, small, and smooth; but *S. thoraltensis* showed no growth. After that, using a Gram stain, the cells were examined microscopically, and their sizes, colors, and shapes were determined by looking at the cells under a light microscope [8,50,51,52].

### 3.3 Demographic and clinical data of acne patients

**Figure 4:**
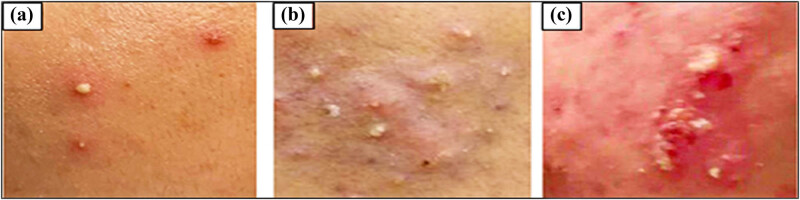
Case displays of acne conditions: (a) subtle, (b) intermediate, and (c) intense.

**Figure 5:**
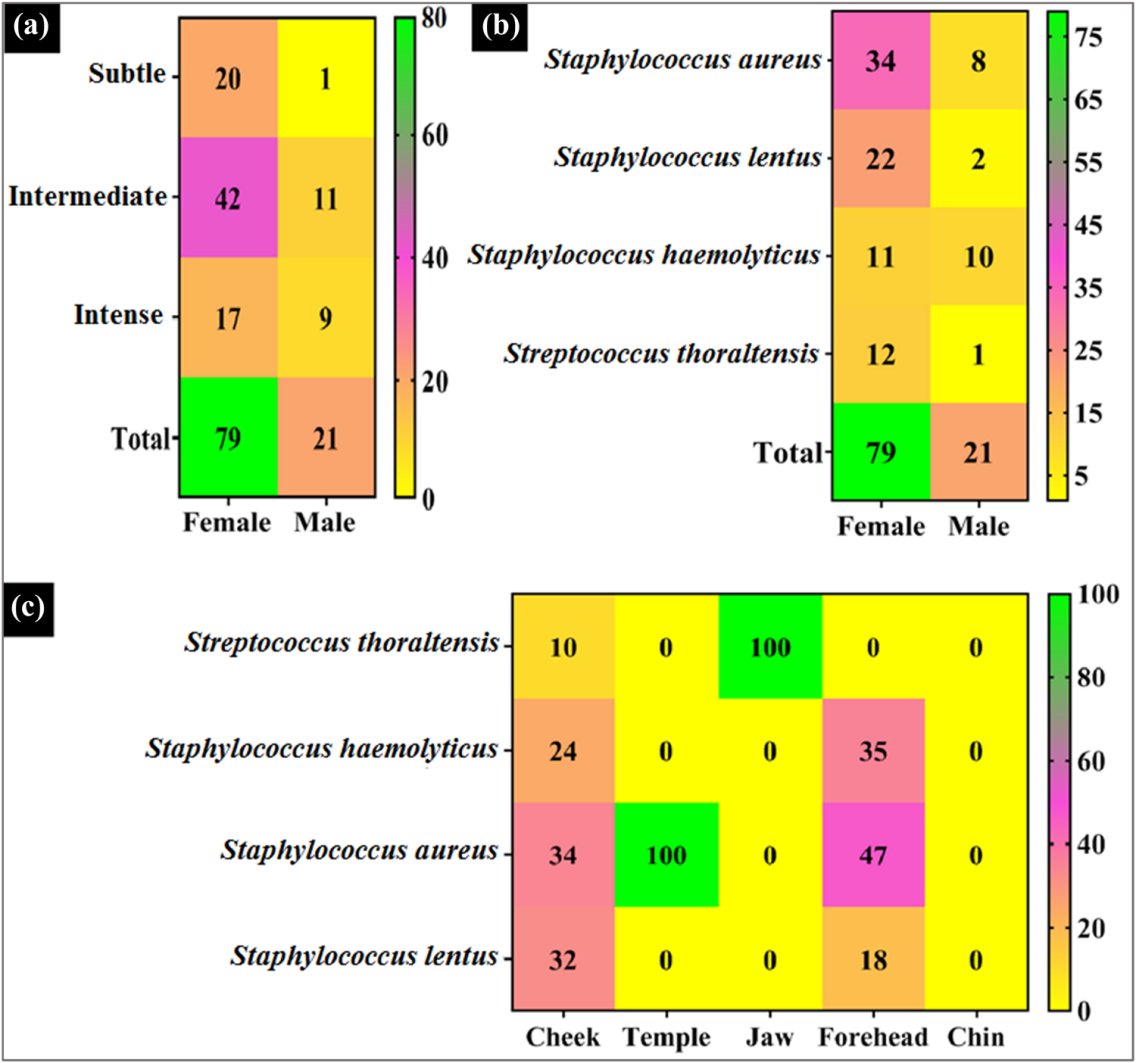
Distribution percentage of (a) acne severity with gender-wise distribution, (b) bacterial acne distribution with gender, and (c) bacterial abundance with different face regions.

Ninety acne patients with mild, moderate, and severe bacterial acne were enrolled in the study (Figure 4). To explore the antibacterial activity potential of GANPs, microorganisms were isolated from these clinical tissues. With 21% in men and 79% in women, acne was more common in women than in men, as seen in Figure 5a. Acne, which is generally caused by a variety of reasons, including emotions, hormone fluctuations (especially in women), various kinds of bacteria presence, environmental factors, and insulin resistance, is more common in women, which increases their risk of developing acne vulgaris at higher rates, as observed. Gruszczyńska et al. [53] recently conducted a study wherein insulin-resistant patients had statistically increased incidences of acne vulgaris. Also, the polycystic ovarian syndrome has been found to be a crucial factor in the development of acne [54]. Acne has been reported to be aggravated by increased exposure to environmental risk factors and unfavorable conditions, such as prolonged perspiration, sun exposure, high temperature, air pollution, and an abundance of halogenated substances. Numerous variables, including stress, poor sleep quality, obesity, and family history, might have a bad impact on acne development and complications [55]. The distribution of bacterial acne by gender (Figure 5b) indicates that *Staph aureus*, a bacterium that is thought to be resistant to drugs, infected more women than men. It is more persistent because of oily and mixed skin types that contain lipase enzymes, which are essential for the colonization and proliferation of acne infectious sites, wherein oily and mixed skin types offer a favorable environment, especially for *Staphylococcus* species [56]. Moreover, a larger percentage of women have adopted new skincare regimens and products without consulting a dermatologist, more frequently after discovering them online, through friends or acquaintances, and have added to the situation. Because of the increased bacterial resistance, the acne gets worse, and according to a recent study, 9.5% of patients who used skincare products found their acne condition worsening [57]. Additionally, as observed, patients in the 15–25-year age range reported the highest acne prevalence. Due to hormonal changes at this age, keratinocytes and sebaceous glands grow and produce excessive amounts of oily sebum, which is the primary cause of acne [58]. Ultimately, the data showed variations in the kinds and quantities of bacterial acne at various locations. The cheek and jaw were found to have the highest prevalence of acne spots and were among the top face-located bacterial isolate sampling sites (Figure 5c). The distribution of *Staph aureus*, *Staph haemolyticus*, *Staph lentus*, and *S. thoraltensis* on different face regions is displayed on the heat map. The diverse range of bacterial species that comprise the skin microbiome serves to shield the host organism and the skin from external agents and other vulnerable microorganisms. On the other hand, an imbalance of opportunistic commensal bacteria resulting from changes in the external environment allows the bacteria to proliferate and causes skin conditions, such as acne vulgaris [59].

### 3.4 Antibacterial activity

#### 3.4.1 Agar well diffusion analysis

**Figure 6:**
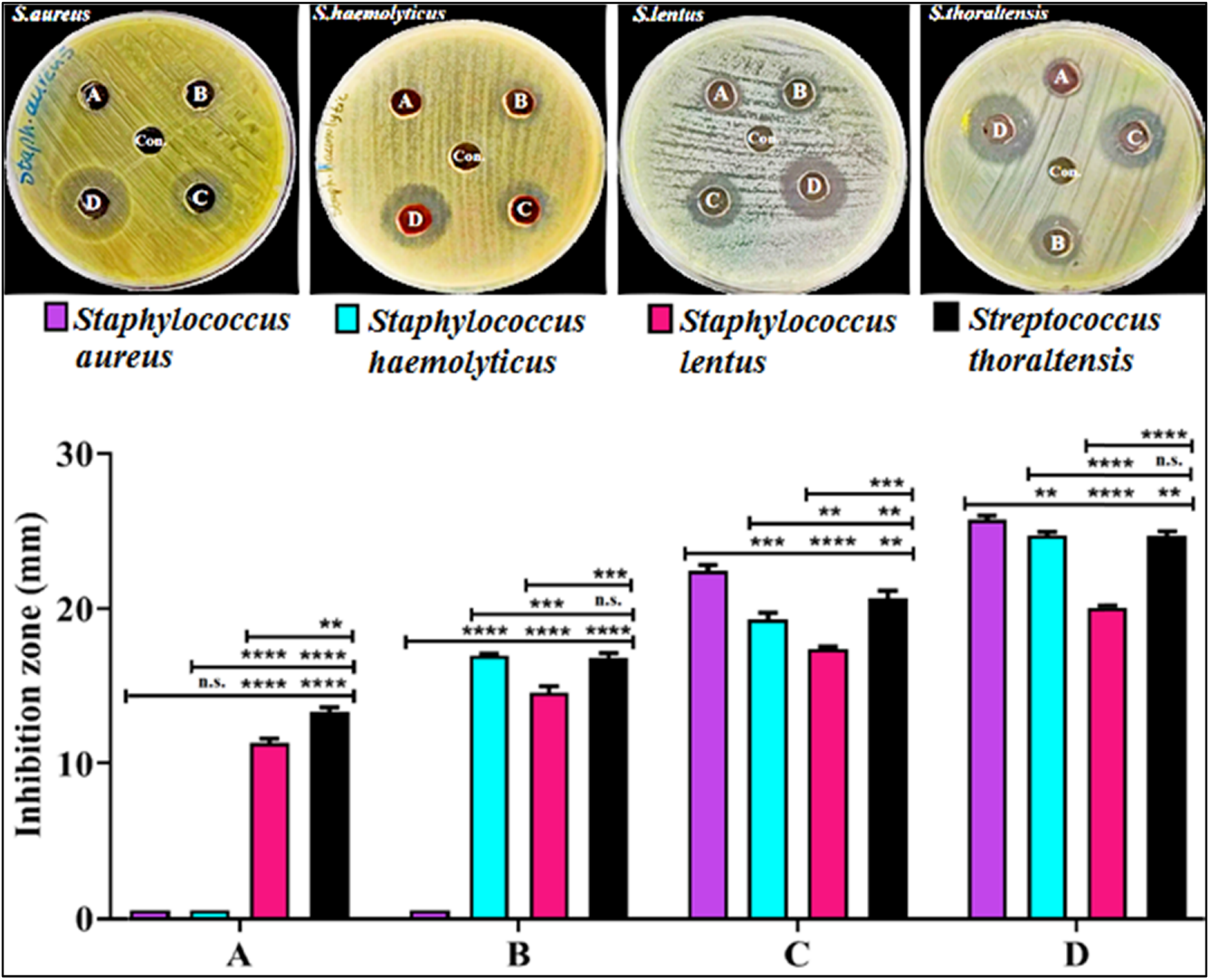
Inhibition zones produced by GANPs against *Staph aureus*, *Staph haemolyticus*, *Staph lentus*, and *S. thoraltensis* at concentrations of (a) 800, (b) 1,600, (c) 3,200, and (d) 6,400 µg/mL, with DW as control. The values are presented as the mean ± SD from three replicate experiments. The n.s. refers to not significant, **p < 0.01, ***p < 0.001, and ****p < 0.0001.

By using the agar well diffusion method, the antibacterial activity of GANPs was assessed at different concentrations (6,400, 3,200, 1,600, and 800 µg/mL), which demonstrated the inhibitory effects of GANPs against *Staph aureus*, *Staph haemolyticus*, *Staph lentus*, and *S. thoraltensis*. Figure 6 shows the observation of substantial inhibition zones following the organisms’ treatment with the prepared GANPs. With an average inhibition zone diameter of 25.74 mm, the *Staph aureus* bacteria tested had the highest inhibitory diameter of the GANPs in this study at 6.4 mg/mL. Next, came the *Staph haemolyticus* bacteria, which had an average inhibition zone diameter of 24.72 mm, and then, the *S. thoraltensis* bacteria, which had an average inhibition zone diameter of 24.71 mm. Finally, the *Staph lentus* bacteria test had the smallest inhibitory diameter, at an average of 20.07 mm at 6,400 µg/mL. *Staph aureus* and *Staph haemolyticus* were able to show low levels of resistance because they could make exotoxins, which are a key part of virulence that makes bacteria more resistant to antimicrobial drugs [60]. Because of their small size, excellent penetration ability, and high specific surface area, GANPs can effectively create a zone for antibacterial action. By interacting electrostatically with the bacterial cell membrane, GANPs can bind to it and disrupt the permeability of the outer membrane, destroying the peptidoglycan layer in the process [61]. After penetrating the inner membrane layer, the GANPs act on bases to damage the DNA. Additionally, peptide substrates on tyrosine residues undergo de-phosphorylation, which inhibits signal-transduction phosphorylation and disables the proton motive forces across the cytoplasmic membrane, ultimately resulting in cell death [62]. Another way that GANPs may work is by inhibiting respiratory enzymes with the gold ions, which block respiratory chain dehydrogenase and dissociate the respiratory chain, causing the release of reactive oxygen species, which results in cell self-attacking [63]. Unlike traditional antibiotics and treatments, which act on microbial cells through only one mechanism, GANPs can exhibit different mechanisms as antibacterial agents, and consequently, the bacterial acne will become incapable of developing resistance to the available GANPs. The GANPs may function as antibacterial agents through a variety of routes, in contrast to conventional antibiotics and therapies that exclusively affect microbial cells through one method. As a result, the bacterial acne is prevented from becoming resistant to GANPs.

#### 3.4.2 MIC analyses

**Figure 7:**
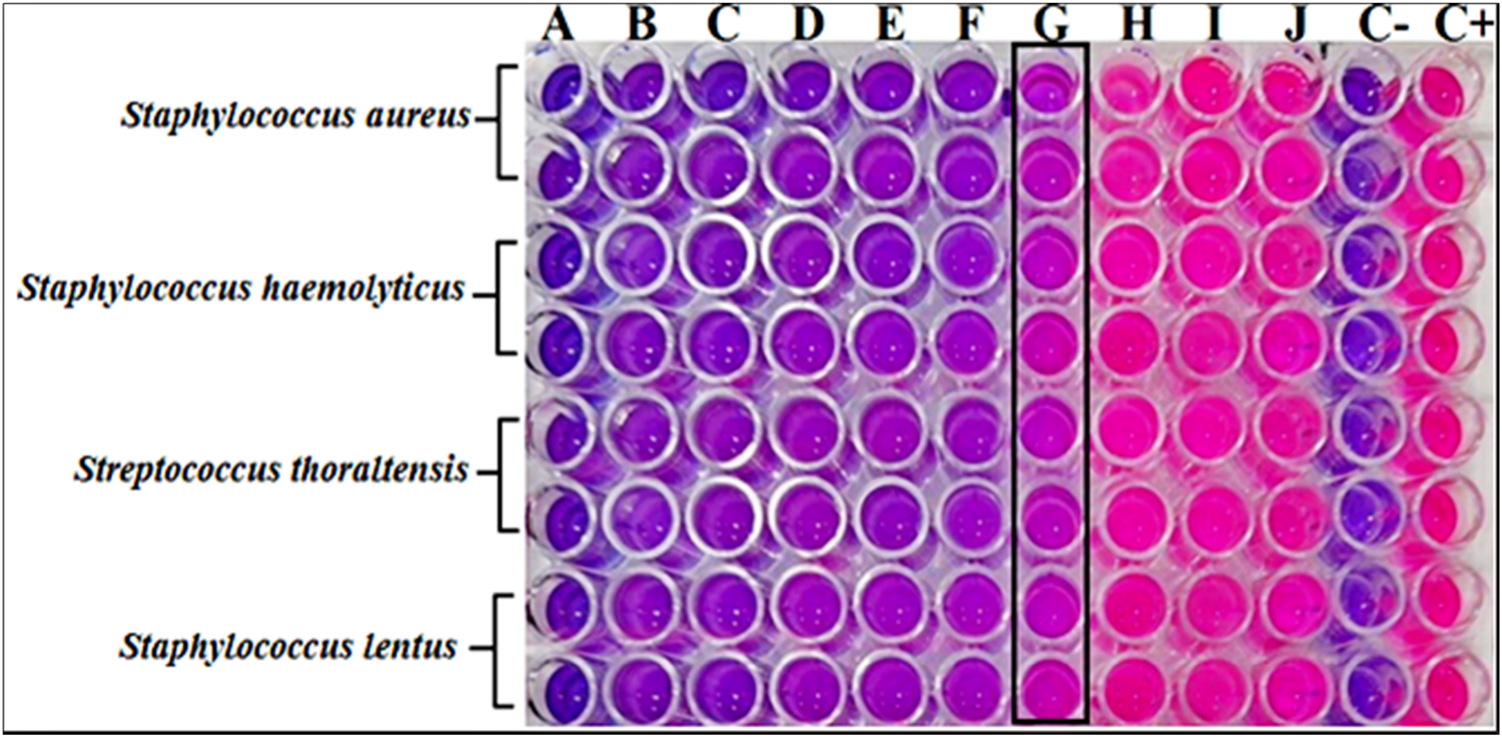
MIC determinations of GANPs against *Staph aureus*, *Staph haemolyticus*, *S. thoraltensis*, and *Staph lentus* at concentrations of (a) 6,400, (b) 3,200, (c) 1,600, (d) 800, (e) 400, (f) 200, (g) 100, (h) 50, (i) 25, and (j) 12.5 µg/mL, and C– as negative control contained only broth medium while C+ as positive control contained broth medium and bacterial suspension.

Using a visual comparison among the wells treated with GANPs and those of the negative and positive controls, the MIC efficacy of GANPs against *Staph aureus*, *Staph haemolyticus*, *S. thoraltensis*, and *Staph lentus* was assessed during 24 h, which indicated excellent antibacterial activity of the GANPs. The MIC activity of GANPs was measured at various concentrations, ranging from 6,400 to 12.5 µg/mL. Resazurin was used in a colorimetric assay method to assess the impact of GANPs on the development of bacteria isolated from acne. In wells containing 6,400–200 µg/mL, GANPs have shown total elimination of the microbes; however, under these concentrations, some bacterial growth was observed in the microplate wells for a brief time at the beginning of the experiment. The MIC of GANPs against *Staph aureus*, *Staph haemolyticus*, *S. thoraltensis*, and *Staph lentus* was at 100 µg/mL, which was indicated by the color shift to a pink hue due to bacterial activity. As seen in Figure 7, the outcomes demonstrated that every acne-isolated bacterium was susceptible to GANPs. These produced GANPs had antibacterial action, presumably as a result of attaching the sulfur-containing proteins in the Gram-positive bacterial cell membranes, generating pits and holes that caused the cell membrane to lyse. Following that, the interaction of GANPs with mitochondria resulted in the inhibition of ATPase function, lowering of ATP levels, leakage of internal cell organelles, damage to DNA, and eventually cell death [64,65]. These results showed that GANPs have significant antibacterial activity against Gram-positive bacterial acne at lower concentrations than those observed in the previous report [66]. This report showed that AuNPs with a particle size of 18–28 nm, synthesized by chitosan as a stabilizing and reducing agent, had MIC values of 125 and 250 µg/mL, respectively, against the species of *Staph aureus*.

The anti-biofilm and anti-adhesion activities of GANPs were tested against *Staph aureus*, *Staph haemolyticus*, *S. thoraltensis*, and *Staph lentus*. The results showed that most isolates were stopped at 100 µg/mL, which was in line with what was seen in the MIC analysis. *S. thoraltensis* demonstrated non-significant change at 100 µg/mL in the anti-adhesion assay, due to the *Sua* gene, which is the designated gene present only in *S. thoraltensis*, playing a crucial role in the formation of strong adhesion [67].

#### 3.4.3 Detection of biofilm formation and anti-biofilm analysis

**Figure 8:**
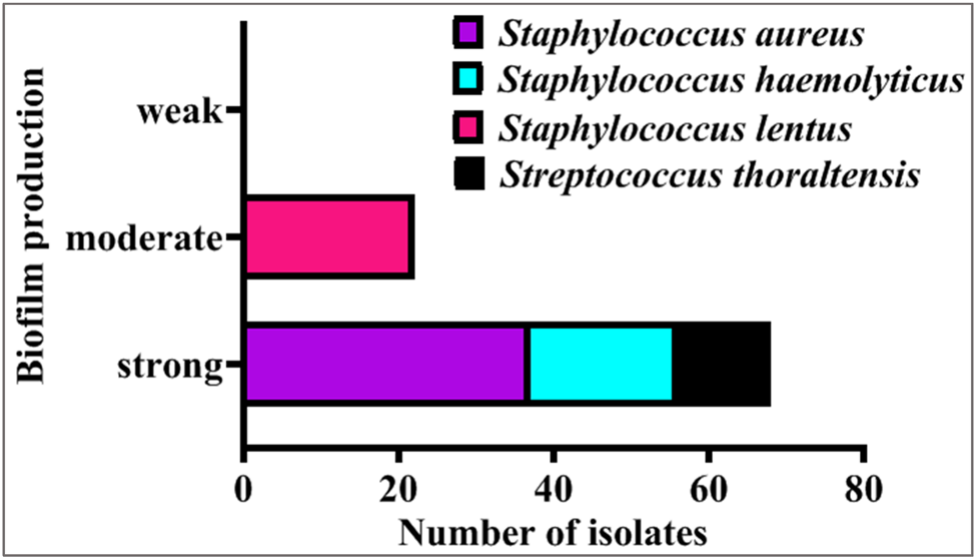
Quantitative screening of biofilm formation by microtiter plate method.

**Figure 9:**
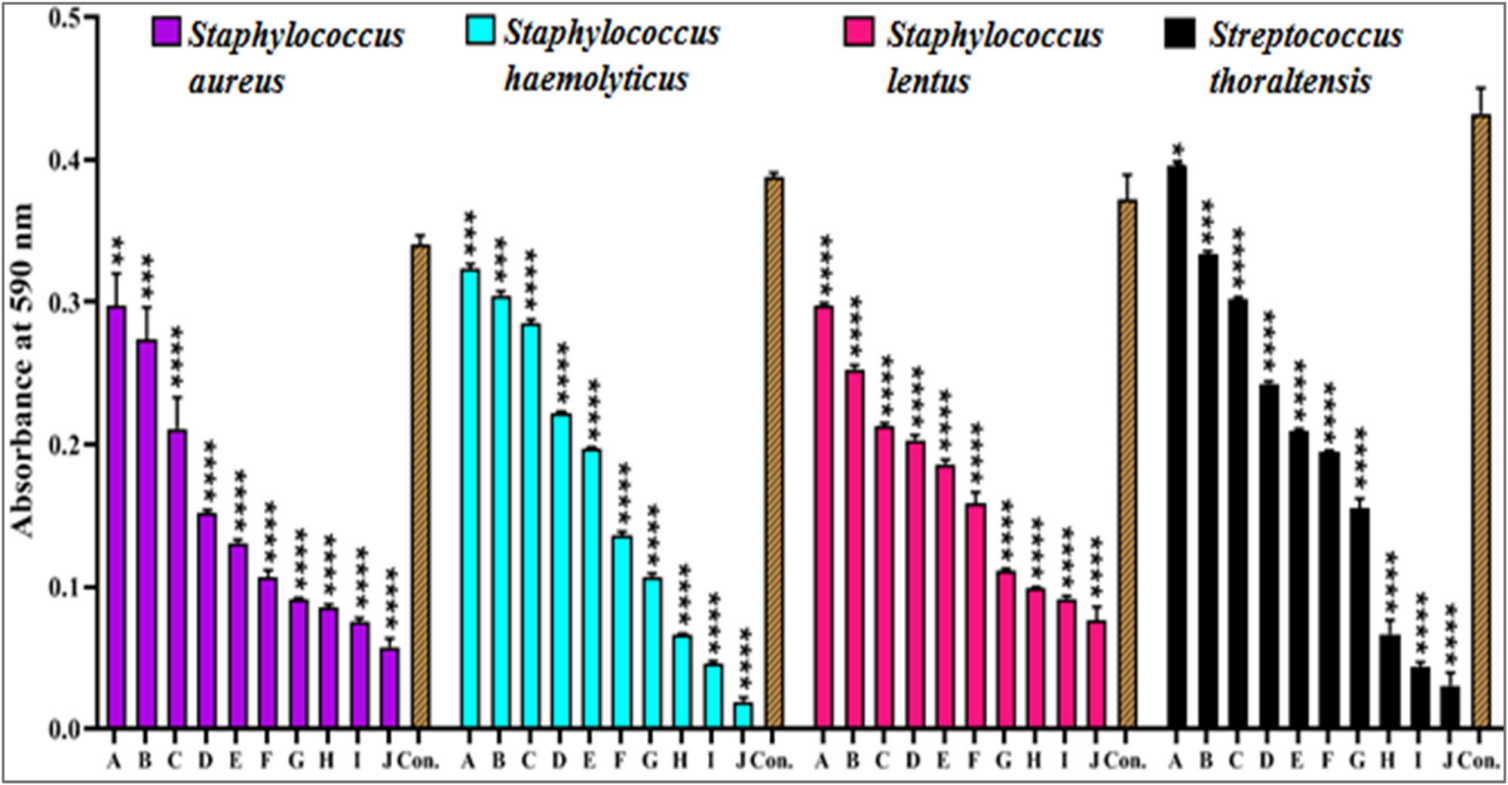
Anti-biofilm activity of GANPs at concentrations of (a) 100, (b) 150, (c) 200, (d) 250, (e) 300, (f) 350, (g) 400, (h) 500, (i) 600, and (j) 700 µg/mL against *Staph aureus*, *Staph haemolyticus*, *Staph lentus*, and *S. thoraltensis*, after incubation for 24 h with the positive control, according to optical density absorption values at 590 nm. The values are presented as the mean ± SD from triplicate experiments. *p < 0.05, **p < 0.01, ***p < 0.001, and ****p < 0.0001.

The microtiter plate method demonstrated that all 90 acne-isolated bacteria could create a strong biofilm whereas the isolates of *Staph lentus* could only form a moderate biofilm [68], as shown in Figure 8. The anti-biofilm activity of GANPs against *Staph aureus*, *Staph haemolyticus*, *Staph lentus*, and *S. thoraltensis* was assessed using a microtiter plate staining procedure with crystal violet. The absorbance measurements were performed by the microplate absorbance reader at 590 nm at different concentrations (100, 150, 200, 250, 300, 350, 400, 500, 600, and 700 µg/mL), and a decrease in the values with increasing GANPs concentration was observed, which indicated that adding the GANPs inhibited and prevented the formation of the biofilm of acne-isolated bacteria (Figure 9).

The GANPs showed a significant change in absorption values for all the bacterial species, from the highest concentration of 700 µg/mL to the lowest. There are several possible explanations for the anti-biofilm effect of GANPs. One of them is that they impede the synthesis of exogenous polysaccharides by entering the water channels, which carry nutrients and water through the layers of polysaccharides found in bacterial cell walls [69,70], suppressing quorum sensing and breakdown of intramolecular forces. Results showed that nanoparticles’ concentration, size, and outreach distribution all had a significant impact on the anti-biofilm activity of the GANPs. Smaller nanoparticles exhibited a higher anti-biofilm activity because their smaller size allowed them to adhere to the bacterial membranes and penetrate the biofilm, interfering with membrane permeability and cellular metabolism [71]. These findings are in line with a prior study [72] showing that higher concentrations (75, 100, and 200 µg/mL) of GANPs affected the generation of biofilms against *Staph aureus* when compared to the lower concentrations. Also, in another study [73], it was found that higher numbers of AuNPs had significant effects on the antibacterial action. A small increase in the AuNPs number inhibited 99.99% of biofilm formation by *Klebsiella* and *Staphylococcus* species.

#### 3.4.4 Anti-adhesion analysis

**Figure 10:**
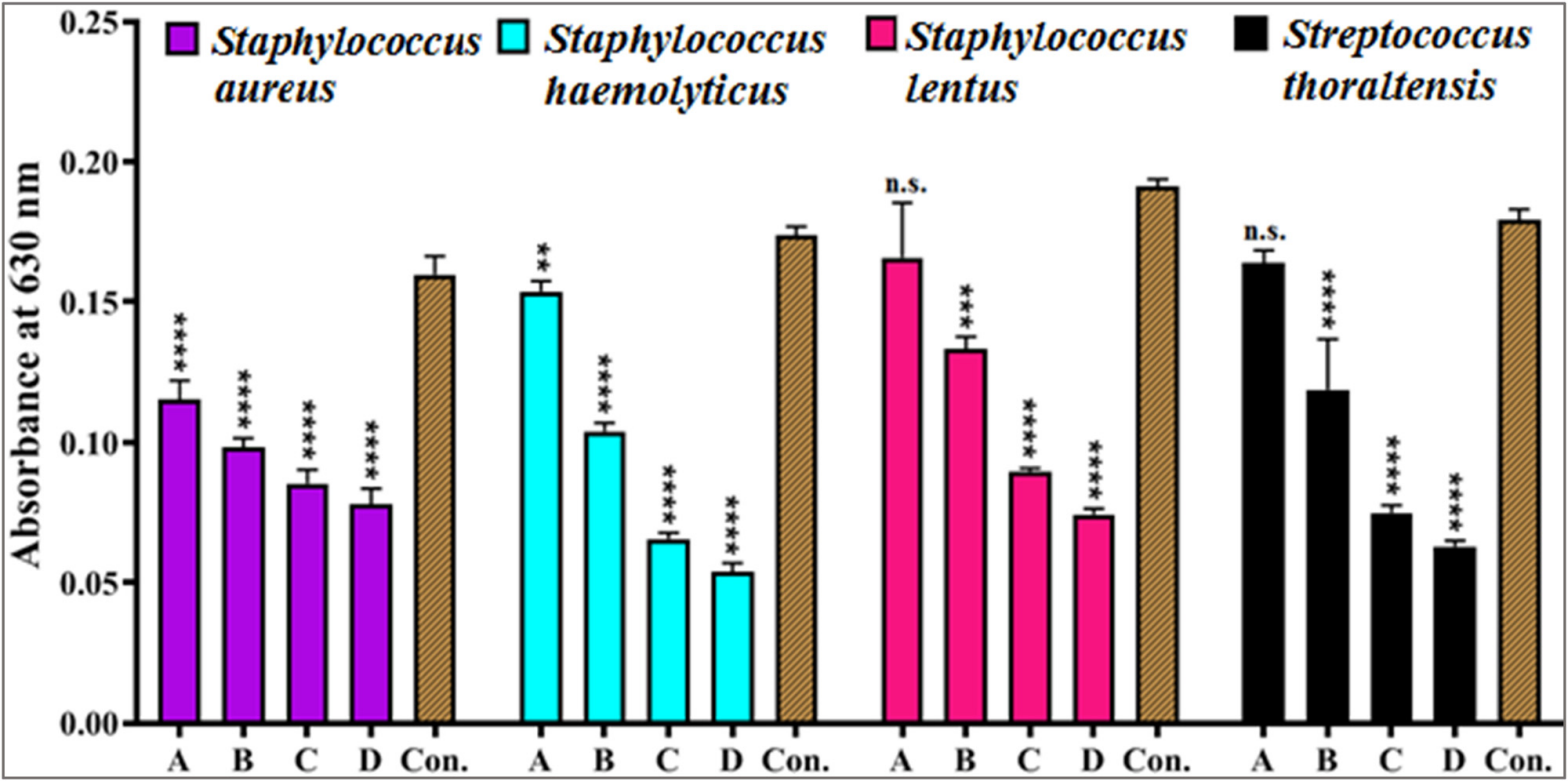
Anti-adhesion assays of GANPs at concentrations of (a) 100, (b) 200, (c) 600, and (d) 700 µg/mL, after incubation for 4 h against *Staph aureus*, *Staph haemolyticus*, *Staph lentus*, and *S. thoraltensis* with positive control, according to optical density values at 630 nm. The values are presented as the mean ± SD. from triplicate experiments. The n.s. refers to not significant, **p < 0.01, ***p < 0.001, and ****p < 0.0001.

The anti-adhesion activity of GANPs against *Staph aureus*, *Staph haemolyticus*, *Staph lentus*, and *S. thoraltensis* was assessed using a microtiter plate staining technique with crystal violet, with measurements made by the microplate absorbance reader at 630 nm. The concentrations of GANPs were 100, 200, 600, and 700 µg/mL, as shown in Figure 10. The acne-isolated bacteria’s initial adherence to the polystyrene microplate was prevented by the addition of GANPs, as seen by the drop in the optical density values as the GANPs concentration increased. The process involved GANPs accumulating beneath the bacterial cells and interacting with the surface once they arrived, thereupon increasing the cell wall tension and interfering with the proteins and enzymes necessary for microbial adhesion [74]. The extracellular polymeric substances, which function primarily as the biofilm’s adhesive material needed for biofilm formation, were not available [75]. Following this, the GANPs either entirely move out or are released as gold ions (Au^+^) into the interior of the biofilm, where they interact with bacterial cells and biofilm components to produce metabolic imbalance and nano-toxicity, which ultimately results in bacterial cell death. Numerous variables, including the size, surface charge, and chemical makeup of the biofilm as well as the shape of the biofilm surface, affect the penetration of nanoparticles. The GANPs’ antibacterial efficacy is contingent upon the disturbances that transpire within distinct bacterial components and biofilms. Hydrophobic interactions, hydrogen bonding, electrostatic, and van der Waals attraction interactions are all involved in biofilm penetration techniques [76,77].

## 4 Conclusions

Bacterial acne is becoming more common and resistant to antibiotics. The GANPs are non-toxic and have smaller sizes, which makes them an attractive alternative material for being used as an antimicrobial agent. The GANPs demonstrated significant antibacterial activity at low concentrations against Gram-positive bacteria sourced from acne. The GANPs damaged bacterial cells through multiple mechanisms, making it difficult for the pathogenic bacteria to develop resistance. The remarkable biological activity of the GANPs presented a viable potential for adopting as a superior skin treatment for severe acne, provided their clinical competence. Also, future studies using animal models are needed to explore the antibacterial efficacy of these prepared nanoparticles in wounds and burns that are contaminated with bacterial species encountered in acne.
